# *Cysticercus fasciolaris* in Brown Rats* (Rattus norvegicus)* in Grenada, West Indies

**DOI:** 10.1155/2017/1723406

**Published:** 2017-11-26

**Authors:** Ravindra Sharma, Keshaw Tiwari, Kristen Birmingham, Elan Armstrong, Andrea Montanez, Reneka Guy, Yvette Sepulveda, Veronica Mapp-Alexander, Claude DeAllie

**Affiliations:** School of Veterinary Medicine, St. George's University, West Indies, Grenada

## Abstract

Cat is the definitive host of* Taenia taeniaeformis (T. taeniaeformis). Cysticercus fasciolaris (C. fasciolaris), *the larval stage of* T*.* taeniaeformis, *develops in small rodents which act as intermediate host. The aim of this study was to estimate the prevalence of* C. fasciolaris *in brown rats* (Rattus norvegicus)* in the densely human populated parishes, St. George's and St. David's of Grenada, West Indies. One hundred and seventy rats were trapped near the residential areas from May to July, 2017 and examined for* C. fasciolaris *in their liver. Of the 170 rats 115 (67.6%, CI 95% from 60.1 to 74.6) were positive for the larval stage of* T. taeniaeformis*. One to three cysts were observed in each liver, containing a single larva in each cyst. The prevalence was 77.9% in St. George and 59.1% in St. David which is a significant difference (*p* < 0.05) between the two parishes under study. Based on gender, prevalence in males was 60.9% and females 74.7%. Significant difference was observed between young and adult rats (*p* = 0.03). Prevalence in young rats was 45.0% compared to adults (70.7%). Further study of risk assessment in the cat population in areas of the present research is strongly suggested.

## 1. Introduction

*Taenia taeniaeformis *is a cestode parasite found in the intestine of cats as final host. Wild rodents, mainly mice, various species of rats, and voles act as intermediate host for the parasite. The intermediate hosts get infected through ingestion of contaminated feed, water, and beddings from eggs of the parasite voided by cats. Eggs develop into larval form (metacestodes) in the liver of intermediate host. The larval form of* T. taeniaeformis *is called* C. fasciolaris. Taenia crassicollis, Hydatigera fasciolaris, Strobilocercus, *and bladder worm are synonyms of* Cysticercus fasciolaris *[1].* C. fasciolaris *develops mainly in the liver of rodents and contains larval stages of the parasite. Occasionally cysts also develop in the abdominal wall and kidney, filled with purulent exudate without larvae [2]. A small number of fibrosarcoma cases in the liver of rats associated with cysts of* T. taeniaeformis *have been reported [3–5]. Cats get infected by ingestion of rodents infected with* C. fasciolaris. *Although rare, humans get infected with eggs of* T. taeniaeformis *from cats [6].

*T. taeniaeformis *has been reported in rodents and cats worldwide. The report of* C fasciolaris* particularly, in brown rats* (R. norvegicus),* is from India [7, 8], Korea [2], Malaysia [9], Serbia [10], and USA [3]. In Grenada, during a survey conducted in 2005 for* Angiostrongylus cantonensis (A. cantonensis)* in lung/heart of* R. norvegicus* [11], lesions of* C. fasciolaris *in the liver of (29.6%) rats were also reported. As far as authors are aware, there is no published report of* C. fasciolaris *in brown rats in other Caribbean nations. The aim of this report is to estimate the prevalence of* C. fasciolaris *in brown rats from Grenada and compare with the previous report.

## 2. Materials and Methods

### 2.1. Ethical Approval

The project (Detection of zoonotic pathogens in brown rats in Grenada) was approved by the Institutional Animal Care and Use Committee (IACUC # 16009-R) of St. George's University Grenada.

### 2.2. Study Area

Grenada is the southernmost country in the Caribbean Sea with an area of 348.5 Km^2^. The country with low hills, small trees and shrubs, and tropical climate is most suitable for the existence of brown rats. The country is divided into six parishes. The parishes of St. George and St. David were selected for sampling because of their dense human population compared to the other four parishes.

### 2.3. Species of Rat

Brown rats or Norway rats* (R. norvegicus) *belong to genus* Rattus *under the family Muridae [12]. They are also called brown rats or sewer rats. Brown rats have stocky, gray brown bodies with shorter tail than body length. Brown rats have prominent and pale ears which stick up above the head. Brown rats are larger than most other rat species [13].

### 2.4. Collection of Rats

One hundred and seventy rats were collected live from 1st May to 14th July, 2017, using live traps (45 cm* l* × 15 cm* w* × 15 cm* h*) with cheese and or various local fruits as bait. Attempts were made to trap the rats near the residential buildings. Trapping in both parishes was conducted near 10-meter periphery of human dwellings. Traps were placed in the evening and visited next day during the morning. Traps with rats were covered with black cloth and transported to the necropsy laboratory of the school of veterinary medicine, St. George's University, Grenada, and transferred to the anesthesia machine. Rats were anesthetized using isoflurane in oxygen via anesthesia machine (portable vet anesthesia machine isoflurane vaporizer VET CE), manufacturer DRE (Avante health Solution Company USA).

### 2.5. Collection of Samples

The anesthetized rats were examined physically for their health and weighed. The abdominal cavity of rats was opened using a surgical blade and a pair of forceps. Liver, lung, kidney, and abdominal cavity were examined and recorded for gross lesions of* C. fasciolaris*. Those tissues with gross lesions were fixed in 10% neutral buffered formalin, processed for paraffin embedding, sectioned at 4 *µ*m thickness, stained with hematoxylin and eosin, and examined under the light microscope. Before fixation of tissues, the parasites were removed from the cysts and examined. Prevalence of infection was calculated as the number of infected animals divided by the number of examined animals.

### 2.6. Statistical Analysis

The data was analyzed by the statistical analysis: Fisher's exact test, using graphical statistical software (https://www.graphpad.com/quickcales/contingency2).

## 3. Results and Discussion

Trapped rats were examined physically for their body condition and signs of illness. Weak and fragile with rough hair coat were the criteria used for illness. No apparent illness was observed in any rat. Previous researchers [10, 14] also reported the healthy physical status of rats in spite of* C. fasciolaris *in their liver.

Out of 170 brown rats examined, 115 showed lesions of* C. fasciolaris* in their liver, giving 67.6% (95% CI from 0.6006 to 0.7461) positivity. The results for the prevalence are included in Table 1. The results showed 77.9% and 59.1% of positive rats in St George's and in St. David's parishes, respectively. Prevalence of* C. fasciolaris* by parish was statistically significant (*p* < 0.05). Risk factors being similar in both parishes, this difference in prevalence is not well explained. Further research involving more number of rats is suggested to answer the difference. Previous researchers reported in brown rats a prevalence of 100% in the Philippines [14], 33.3% in India [15], 33.8% in Korea [2], and 29.9% in Serbia [10]. During a study conducted by Chikweto et al. [11] in Grenada on* A. cantonensis* in brown rats, researchers found 29.6% rats also infected with* C. fasciolaris*. Variations in the prevalence of* C. fasciolaris* in different countries indicate infection risk factors, including seasonal variation in the infection pressure on the intermediate hosts [16]. Prevalence rate found in the present report in Grenada is higher compared to previous finding [11]. Since there is not much variation of the season in Grenada, the higher prevalence found in our study could be the result of the sampling areas in our study. Our samples were obtained from two densely human populated parishes, compared to previous study where samples were from all 6 parishes of the country.

**Figure 1:**
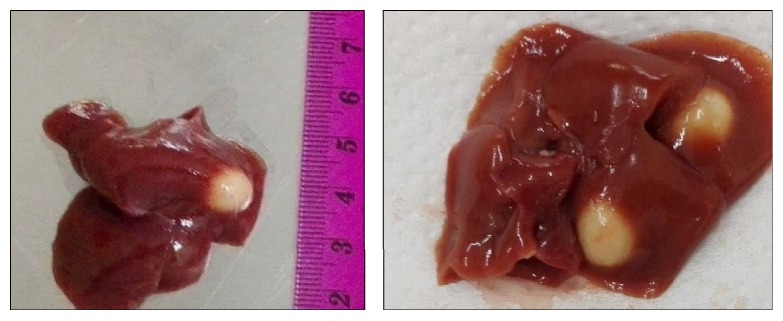
Multiple cysts of* C. fasciolaris *in liver.

**Figure 2:**
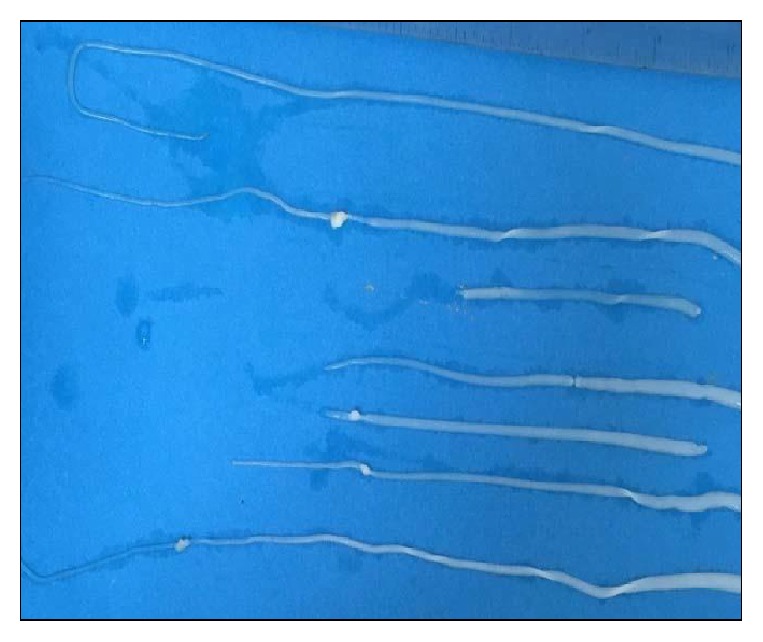
The* Cysticercus fasciolaris *taken out from the cysts.

On gross examination of liver of infected rats, one to three cysts were found in each liver (Figure 1). Size of cysts varied from 2.0 mm to 8.0 mm. Color of the cysts ranged from white to grayish white. Each cyst contained single larvae embedded in white turbid color fluid. Larvae were removed from the cyst to study its characteristics. The usual size of the larvae in the present study varied between 6 and 20 cm (Figure 2) but may reach up to 32 cm [17]. Jithendran and Somvanshi [1] in an experimental study showed that the size of the cyst and larvae vary with their stage of development.

**Figure 3:**
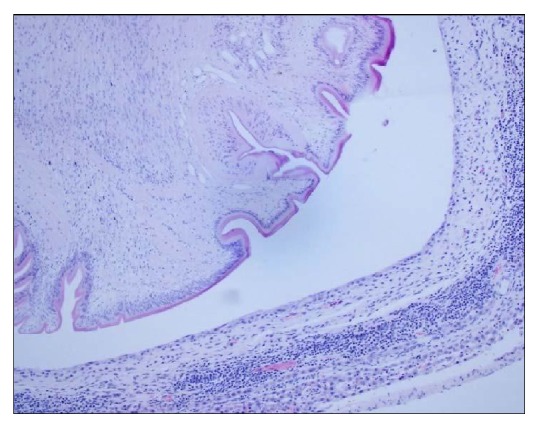
Section of liver of* Rattus norvegicus *showing thick capsule with mononuclear cell Infiltration around a* Cysticercus fasciolaris.*

Histopathology of the liver showed minimal pathological lesion in the liver parenchyma, except in and around the cysts. The cysts had a central lumen which contained* C. fasciolaris*. The wall of cysts varied in thickness from thin connective tissue capsule in mature* C. fasciolaris* and thick wall of connective tissue in juvenile* C. fasciolaris*. These findings are consistent with Lee et al. [2]. Similar to Jithendran and Somvanshi [1] we also found lymphocytic cuffing around the cysts (Figure 3).

The prevalence of* C. fasciolaris* according to gender in the present study is included in Table 2. We report prevalence of* C. fasciolaris* in 66.7% male and 89.5% female in St George's parish compared to 56.3% male and 62.2% female in St David's parish. In our study, there was no significant statistical difference between male and female. Lee et al. [2] and Kataranovski et al. [10] also reported no difference in prevalence of* C. fasciolaris* among male and female rats. However, contrary to our findings Rodríguez-Vivas et al. [18] found higher prevalence in adult male rats. The authors did not explain the reasons for higher prevalence in adult males.

The results for prevalence of* C. fasciolaris* in young and adult rats are tabulated in Table 3. The age of the animals was determined on their weight and size. Rats below 100 G were grouped as young and over 100 G as adult following the methodology used by previous researchers [16, 19]. The prevalence was 45% in young rats and 70.7% in adult rats. This demonstrates a higher rate of infection in adult rats. The difference in prevalence with age groups was statistically significant (*p* < 0.05). Our observation is in accordance with previous researchers [2, 16, 18]. The reason for higher prevalence in adult rats is not well explained. However, Lee et al. [2] indicated that positivity in adults may be reflecting the accumulation of infection with age. To answer this question further research is suggested.

The Grenadian community likes cats as a pet. However, these pets are not always confined inside the home resulting in roaming behavior near and around the residential areas. The population of cats in the study areas of St. George's and St. David is not known. Since rats are the intermediate host and are the final host for* T. taeniaeformis*, there is a need of risk assessment of the rat as well as cat population in these two parishes. This study has found strong evidence to educate the community regarding proper maintenance of hygienic conditions in and around their dwellings to prevent the survival and proliferation of the rat population.

## Figures and Tables

**Table 1:** Prevalence of *Cysticercus fasciolaris *in brown rats of Grenada.

Parish	Number of rats examined	Number of rats infected	Percentage (%) of rats infected
St. Georges	77	60	77.9%^*∗*^
St. David	93	55	59.1%^*∗*^
Total	170	115	67.6%

^*∗*^ *p* value equals 0.0132.

**Table 2:** Prevalence of *Cysticercus fasciolaris *in brown rats of Grenada according to gender.

Parish	Male		Female	
	Number of rats examined	Number of rats infected (%)	Number of rats examined	Number of rats infected (%)
St. Georges	39	26 (66.7%)	38	34 (89.5%)
St. David	48	27 (56.3%)	45	28 (62.2%)
Total	87	53 (60.9%)^∗^	83	62 (74.7%)^*∗*^

^*∗*^ *p* value equals 0.0711.

**Table 3:** Prevalence of *Cysticercus fasciolaris *in brown rats of Grenada according to age.

Parish	Young		Adult	
	Number of rats examined	Number of rats infected	Number of rats examined	Number of rats infected
St. Georges	13	9 (69.2%)	64	51 (79.7%)
St. David	7	0 (0.0%)	86	55 (64.0%)
*Total*	*20*	*9 (45.0%)* ^∗^	*150*	*106 (70.7%)* ^∗^

^*∗*^ *p* value equals 0.0389.
